# Complete Genome Sequence of *Xanthomonas* Siphophage Samson

**DOI:** 10.1128/MRA.01097-19

**Published:** 2019-10-17

**Authors:** Sabrina Clark, Tram Le, Russell Moreland, Mei Liu, Carlos F. Gonzalez, Jason J. Gill, Jolene Ramsey

**Affiliations:** a Center for Phage Technology, Texas A&M University, College Station, Texas, USA; Loyola University Chicago

## Abstract

The Xanthomonas genus includes many Gram-negative plant-associated bacteria. Here, we report a virulent *Xanthomonas* siphophage called Samson. A siphophage isolated from sewage, Samson contains a 43,314-bp genome with 58 predicted genes. Samson has high nucleotide identity with Pseudomonas phage PaMx42.

## ANNOUNCEMENT

The Xanthomonadaceae are a diverse family of plant-associated Gram-negative bacteria (1). Some species within both the *Xanthomonas* and Xylella genera result in devastating diseases among important food crops (2). Recent efforts using bacteriophage application to mitigate Pierce’s disease caused by Xylella fastidiosa in grape vines were successful (3). Several broad-host-range phages infecting both *Xylella* and *Xanthomonas* species have been reported (4) and provided the motivation for our discovery of Samson, the phage reported here.

Bacteriophage Samson was isolated from filtered (pore size, 0.2 μm) wastewater samples collected in College Station, Texas. Samson was propagated by the soft-agar overlay method of Adams (5) on a rice isolate of *Xanthomonas* (ATCC PTA-13101), as described by Ahern et al. (4). Morphology was acquired by negative staining of phage samples with 2% (wt/vol) uranyl acetate and viewing with transmission electron microscopy at the Texas A&M Microscopy and Imaging Center (6). Genomic DNA was purified by the shotgun library modification of the DNA Wizard kit (Promega) reported by Summer (7). An Illumina TruSeq paired-end 250-bp library was prepared with the Nano low-throughput kit for sequencing on the Illumina MiSeq platform using the v2 500-cycle chemistry. Quality control was performed on the 414,121 total reads with the FastQC method (https://www.bioinformatics.babraham.ac.uk/projects/fastqc/). After trimming with the FastX Toolkit v0.0.14 (http://hannonlab.cshl.edu/fastx_toolkit/), the phage genome was assembled into a single contig with SPAdes v3.5.0 (using default parameters) at a coverage of 182.7-fold (8). The raw contig was confirmed complete by Sanger sequencing of a PCR product amplified off the contig ends (forward, 5′-TGTGATCGGTCTTGCTGAAATC-3′; reverse, 5′-CACCTGTTCGCCCTTCTT-3′). Gene calls were made based on analyses with GLIMMER v3.0 and MetaGeneAnnotator v1.0, while tRNA genes were detected by ARAGORN v2.36 (9–11). Putative terminators (rho independent) were annotated from TransTermHP v2.09 (12). Gene functions were then predicted using searches for conserved domains with InterProScan v5.33-72 and by similarity searches with a 0.001 maximum expectation value cutoff in the NCBI nonredundant database and the UniProtKB Swiss-Prot/TrEMBL databases by BLAST v2.2.31 (13–15). Potential transmembrane domains were inspected with TMHMM v2.0 (16). Structural predictions were done with the HHSuite v3.0 HHPred tool (multiple sequence alignment [MSA] generation with HHblits using the ummiclus30_2018_08 database and modeling with the PDB_mmCIF70 database) (17). The genomic terminus type was assigned from PhageTerm analysis (18). Whole-genome sequence similarity alignments were carried out by the progressiveMauve v2.4.0 algorithm (19). All tools listed above were executed with default parameters, unless otherwise stated. Access to these tools (with the exception of HHPred) is provided through the Center for Phage Technology Galaxy and Apollo instances, hosted at https://cpt.tamu.edu/galaxy-pub/ (20, 21).

Samson is a 43,314-bp siphophage with a G+C content of 54.47%. The 94.94% coding density derives from 58 predicted protein-coding genes. PhageTerm predicts that Samson uses a headful-type packaging mechanism. The most closely related phage to Samson, with 95.9% nucleotide identity, is *Pseudomonas* phage PaMx42 (GenBank accession no. JQ067092), with which it shares 56 similar proteins.

### Data availability.

The genome sequence and associated data for phage Samson were deposited under GenBank accession no. MN062187, BioProject no. PRJNA222858, SRA no. SRR8892199, and BioSample no. SAMN11411460.
